# Simple Bedside Muscle‐Related Indicators Improve the Identification of Reduced Kidney Function in Hospitalized Older Adults: A Multicenter Cross‐Sectional Study

**DOI:** 10.1111/ggi.70851

**Published:** 2026-09-24

**Authors:** Shigemi Morishita, Ken‐ei Sada, Masataka Kudo, Naofumi Dobashi, Kiichiro Fujisaki, Sho Sasaki

**Affiliations:** ^1^ Department of Surgery Oida Hospital Sukumo Japan; ^2^ Department of Clinical Epidemiology Kochi Medical School Nankoku Japan; ^3^ Division of Hospital and General Medicine, Department of General Internal Medicine Iizuka Hospital Iizuka Japan; ^4^ Department of Internal Medicine Kochi Prefectural Hata‐Kenmin Hospital Sukumo Japan; ^5^ Department of Nephrology Iizuka Hospital Fukuoka Japan; ^6^ Department of General Medicine, Iwase Satellite for Teaching and Research (STAR) Fukushima Medical University Fukushima Japan; ^7^ Center for Innovative Research for Communities and Clinical Excellence (CiRC2LE) Fukushima Medical University Fukushima Japan; ^8^ Section of Clinical Epidemiology, Department of Community Medicine, Graduate School of Medicine Kyoto University Kyoto Japan

**Keywords:** cystatin C, estimated glomerular filtration rate, kidney function, muscle‐related indicators, older adults

## Abstract

**Aim:**

Creatinine‐based estimated glomerular filtration rate (eGFRcr) may overestimate kidney function in older adults with reduced muscle mass. Although current guidelines recommend cystatin C when creatinine‐based estimates are unreliable, its routine use remains limited. We evaluated whether incorporating simple bedside muscle‐related indicators into eGFRcr improves the identification of reduced kidney function defined by cystatin C‐based estimated glomerular filtration rate (eGFRcys).

**Methods:**

We conducted a multicenter cross‐sectional study of hospitalized adults aged ≥ 60 years at four Japanese community hospitals. Three models were evaluated: eGFRcr only (Basic model), eGFRcr plus calf circumference (Model 1), and eGFRcr plus the finger‐ring test (Model 2). The primary outcome was continuous eGFRcys, and eGFRcys < 45 mL/min/1.73 m^2^.

**Results:**

Among 98 participants (median age 79 years), adding muscle‐related indicators modestly improved model performance. Adjusted *R* ^2^ increased from 0.641 (Basic model) to 0.668 (Model 1) and 0.680 (Model 2). The proportion of participants correctly classified with eGFRcys < 45 mL/min/1.73 m^2^ increased from 56.1% with the eGFRcr alone to 80.5% with both extended models. Discrimination also improved (area under the curve: 0.882 vs. 0.921 and 0.918, respectively), although the differences were not statistically significant.

**Conclusion:**

Incorporating simple bedside muscle‐related indicators to creatinine‐based assessment improved the identification of reduced kidney function defined by eGFRcys in hospitalized older adults. These measures may complement eGFRcr interpretation when cystatin C measurement is unavailable or delayed. External validation is required before clinical implementation.

## Graphical abstract

Adding simple bedside muscle‐related indicators (calf circumference or the finger‐ring test) to creatinine‐based kidney function assessment improved the identification of reduced kidney function defined by cystatin C in hospitalized older adults. These measures may complement interpretation of eGFRcr in routine clinical practice.

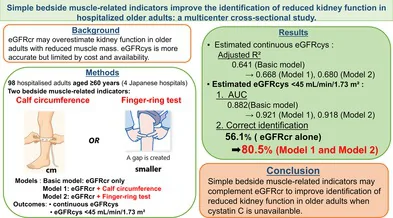

## 1 Introduction

The accurate assessment of kidney function is essential in clinical nephrology, particularly for medication dosing and risk stratification. Impaired kidney function alters the pharmacokinetics of many commonly prescribed drugs, and inappropriate dosing may increase the risk of drug toxicity or therapeutic failure. A systematic review reported that inappropriate prescriptions in patients with renal impairment occurred in 13%–43% of cases [1]. Creatinine‐based estimated glomerular filtration rate (eGFRcr) is the most widely used measure; however, its accuracy is substantially influenced by skeletal muscle mass [2, 3]. In older adults with reduced muscle mass, eGFRcr may overestimate kidney function, potentially masking clinically relevant renal impairment [4, 5]. This limitation may contribute to the under‐recognition of patients who require dose adjustment or closer monitoring.

Cystatin C‐based glomerular filtration rate (eGFRcys) has emerged as an alternative marker that is less affected by muscle mass and provides more reliable estimates in populations with reduced creatinine generation [6]. The KDIGO (Kidney Disease: Improving Global Outcomes) guidelines recommend confirmatory assessment using cystatin C when eGFRcr is likely to be inaccurate, particularly in patients with reduced muscle mass [7]. This recommendation is supported by evidence from the Berlin Initiative Study, in which eGFRcys showed closer agreement with iohexol‐measured GFR than eGFRcr among older adults with discordant creatinine‐ and cystatin C‐based estimates [8]. However, its use remains limited in routine clinical practice due to cost, variability, and reimbursement issues [9, 10, 11]. Therefore, pragmatic and low‐cost approaches are needed to improve the interpretation of eGFRcr without requiring additional laboratory testing or specialized equipment.

Several approaches have been proposed to account for the influence of muscle mass on eGFRcr; however, most are difficult to implement in routine clinical practice. Previously reported methods often rely on specialized techniques such as dual‐energy X‐ray absorptiometry (DXA) [4], computed tomography (CT) [12, 13], or bioelectrical impedance analysis (BIA) [14, 15], which are often impractical for routine clinical use. Simple bedside indicators may be more feasible. Anthropometric measures such as calf circumference [15, 16] have been incorporated into previous prediction models. However, these models have generally combined multiple clinical variables. The finger‐ring test has also been proposed as a rapid, equipment‐free tool to assess reduced muscle mass [17]. Whether these simple bedside indicators can improve the clinical identification of patients whose kidney function is overestimated by eGFRcr remains uncertain.

Therefore, in this multicenter cross‐sectional study, we evaluated whether incorporating simple bedside muscle‐related indicators into creatinine‐based assessment improves the identification of reduced kidney function, defined by eGFRcys, in hospitalized older adults.

## 2 Methods

### 2.1 Study Design and Participants

We conducted a multicenter cross‐sectional study in four community hospitals in Japan between November 2023 and November 2025. This study followed the Transparent Reporting of a multivariable prediction model for Individual Prognosis Or Diagnosis (TRIPOD) statement [18].

Hospitalized patients were enrolled to ensure accurate 24‐h urine collection under nursing supervision. Eligible participants were adults aged ≥ 60 years with an Eastern Cooperative Oncology Group (ECOG) Performance Status ≤ 2 who were able to undergo the finger‐ring test. Patients with indwelling urinary catheters were eligible if they met the same performance criteria. Exclusion criteria were: (i) use of medications affecting creatinine metabolism (trimethoprim, cimetidine, spironolactone, or probenecid); (ii) severe lower‐extremity edema, defined as a persistent indentation lasting > 15 s after finger pressure [19]; (iii) serum creatinine (sCr) ≥ 2.0 mg/dL; and (iv) 24‐h urine volume < 500 mL [20]. The upper sCr threshold was specified to focus on older adults in whom kidney function might be underestimated, rather than those with advanced kidney disease.

The study protocol was approved by the Institutional Ethics Review Board of Kochi University (Approval No. 2023‐95), and written informed consent was obtained from all participants.

### 2.2 Laboratory Measurements and Muscle‐Related Indicators

At all participating hospitals, sCr was measured using a standardized enzymatic assay and serum cystatin C (sCysC) using a particle‐enhanced turbidimetric immunoassay calibrated to an international reference standard. Both biomarkers were measured from blood samples obtained during the same blood draw. eGFRcr and eGFRcys were calculated using equations validated in the Japanese population [21]. Twenty‐four‐hour creatinine clearance (CCr_24_) was calculated as follows:

$$\begin{array}{cc} {\mathrm{CCr}}_{24}\;\left(\mathrm{mL}/\text{min}/1.73\;m^{2}\right)=\; & \left(\text{urine}\;\mathrm{Cr}\times \text{total}\;24\mathrm{‐h}\;\text{urine volume}\;\left[\mathrm{mL}\right]\right)\\ & /\left(\mathrm{sCr}\times 1440\right)\times \left(1.73/\text{body surface area}\;\left[m^{2}\right]\right)\\ & \times 0.715 \end{array}$$

Body surface area was calculated using the Du Bois formula [22]. A correction factor of 0.715 accounts for the systematic overestimation of GFR by creatinine clearance [23].

Lower leg muscle‐related indicators were assessed using two simple bedside measures selected as practical surrogate indicators of muscle mass. Calf circumference was measured at maximal circumference (cm) with participants seated. The finger‐ring test was performed by asking participants to encircle the widest part of the calf using the thumb and index fingers of both hands [17, 24]. Results were categorized as “Smaller” (a visible gap between the fingers) or “Just fit or Larger” (calf circumference equal to or larger than the finger ring). Participants classified as “Smaller” were considered more likely to have reduced muscle mass. All measurements were performed by three trained researchers.

The primary outcome was **c**ontinuous eGFRcys, elected because current KDIGO recommendations support the use of cystatin C when creatinine‐based estimates are likely to be inaccurate because of reduced muscle mass [7]. The secondary outcome was continuous CCr_24_. Dichotomised eGFRcys and CCr_24_ were defined as < 45 mL/min/1.73 m^2^. We selected the threshold of 45 mL/min/1.73 m2 because CKD stage G3b represents a clinically important level at which medication dosing and referral to a nephrologist become increasingly relevant [21].

### 2.3 Statistical Analysis

Continuous variables are presented as medians (interquartile ranges [IQRs]) and categorical variables as numbers (percentages).

Scatter plots and Pearson correlation coefficients were used to examine linearity before model development. Three multivariable linear regression models were developed for continuous outcomes (eGFRcys and CCr24): a Basic model including eGFRcr only, Model 1 including eGFRcr and calf circumference, and Model 2 including eGFRcr and the finger‐ring test. Regression coefficients (*β*), 95% confidence intervals (CIs), and *p* values were reported. Model performance was evaluated using adjusted *R* ^2^ and root mean square error (RMSE). Calibration was assessed using calibration plots, calibration slope, and calibration‐in‐the‐large (CITL).

For dichotomised outcomes (< 45 mL/min/1.73 m^2^), logistic regression models were constructed using the same estimators. Odds ratios (ORs), 95% CIs, and *p* values were reported. Discrimination was assessed using the receiver operating characteristic (ROC) curves and area under the curve (AUC). The DeLong test compared each extended model with the Basic model. Calibration was evaluated using optimism‐corrected calibration slopes and Brier scores based on 1000 bootstrap resamples. Cross‐classification analyses were performed to evaluate the clinical classification performance of each model by comparing estimated kidney function categories with the observed eGFRcys‐ and CCr_24_‐ defined categories. The purpose of these analyses was to examine clinical classification rather than risk reclassification. Internal validation was performed using 1000 bootstrap resamples.

A formal a priori sample size calculation was not performed because reliable assumptions regarding model performance were unavailable. The events‐per‐variable (EPV) values were 20.5 (41 events, two estimators) for the primary outcome and 23.0 (46 events, two estimators) for the secondary outcomes. Because EPV alone does not determine sample adequacy for prediction models [25], optimism‐corrected performance measures are also reported [26].

Statistical significance was defined as *p* < 0.05. All analyses were performed using Stata (version18.0; StataCorp, College Station, TX, USA) and R version 4.4.1 (R Foundation for Statistical Computing, Vienna, Austria).

## 3 Results

### 3.1 Participant Characteristics

Of the 106 hospitalized patients initially enrolled, eight were excluded: four had a 24‐h urine volume < 500 mL, one had a sCr level ≥ 2.0 mg/dL, and three had missing sCysC measurements. The final analysis included 98 participants. Their median age was 79 years (IQR, 73–85), and 60 (61%) were female. The two most common reasons for admission were orthopedic conditions (50.0%) and infectious diseases (23.5%). Median body mass index was 21.8 kg/m^2^ (IQR 19.0–24.9), and median body surface area was 1.48 m^2^ (IQR 1.37–1.60) (Table 1).

**TABLE 1:** Participant characteristics.

	Median (IQR)
Age, years	79 (73–85)
Female, *n* (%)	60 (61)
Reasons for admission, *n* (%)	
Orthopedic conditions	49 (50.0)
Infectious diseases	23 (23.5)
Cerebrovascular diseases	6 (6.1)
Cardiovascular diseases	4 (4.1)
Diabetes‐related Conditions	3 (3.1)
Cancers	3 (3.1)
Others	10 (10.2)
BMI, kg/m^2^	21.8 (19.0–24.9)
Body surface area, m^2^	1.48 (1.37–1.60)
sCr, mg/dL	0.88 (0.69–1.04)
sCysC, mg/L	1.28 (1.05–1.55)
Urine Cr, mg/dL	52.03 (35.81–73.39)
Urine volume, ml	1225 (1000–1800)
eGFRcr, mL/min/1.73m^2^	57.6 (44.4–71.6)
eGFRcys, mL/min/1.73m^2^	49.7 (36.4–61.1)
CCr_24_, mL/min/1.73m^2^	46.4 (35.0–64.7)
Calf circumference, cm	31.0 (28.0–33.0)
Finger‐ring test, *n* (%)	
Smaller	62 (63.3)
Just fit or larger	36 (36.7)

Abbreviations: BMI, body mass index; CCr_24_, 24‐h creatinine clearance; eGFRcr, creatinine‐based estimated glomerular filtration rate; eGFRcys, cystatin‐C‐based estimated glomerular filtration rate; IQR, interquartile range; sCr, serum creatinine; sCysC, serum cystatin C.

### 3.2 Kidney Function and Muscle‐Related Indicators

Median sCr and sCysC levels were 0.88 mg/dL (IQR 0.69–1.04) and 1.28 mg/L (IQR 1.05–1.55), respectively. Median eGFRcr was 57.6 mL/min/1.73 m^2^ (IQR 44.4–71.6), whereas the median eGFRcys was 49.7 mL/min/1.73 m^2^ (IQR 36.4–61.1). Median CCr_24_ was 46.4 mL/min/1.73 m^2^ (IQR 35.0–64.7). Median calf circumference was 31.0 cm (IQR 28.0–33.0). The finger‐ring test classified 62 participants (63.3%) as “Smaller” and 36 (36.7%) as “Just fit or Larger”.

### 3.3 Model Development

All candidate estimators showed approximately linear relationships with continuous outcomes. We developed three multivariable linear regression models for eGFRcys.

$$\begin{array}{c} \text{Basic model}\;\left(\text{eGFRcr only}\right):\text{Estimated eGFRcys}\\ =5.68\;\left(95\%\mathrm{CI}-1.52\;\text{to}\;12.87\right)+0.77\;\left(0.66\;\text{to}\;0.88\right)\\ \;\times \text{eGFRcr} \end{array}$$

$$\begin{array}{c} \text{Model}\;1\;\left(\text{eGFRcr}+\text{Calf circumference}\right):\text{Estimated eGFRcys}\\ =-25.36\;\left(95\%\mathrm{CI}-46.92\;\text{to}-3.80\right)+0.77\;\left(0.66\;\text{to}\;0.88\right)\\ \;\times \text{eGFRcr}+1.00\;\left(0.34\;\text{to}\;1.66\right)\times \text{Calf circumference}\;\left(\mathrm{per}\;1\;\mathrm{cm}\right) \end{array}$$

$$\begin{array}{c} \text{Model}\;2\;\left(\text{eGFRcr}+\text{Finger}-\text{ring test}\right):\text{Estimated eGFRcys}\\ =10.74\;\left(95\%\mathrm{CI}\;3.40\;\text{to}\;18.07\right)+0.77\;\left(0.66\;\text{to}\;0.88\right)\\ \;\times \text{eGFRcr}-8.40\;\left(-13.02\;\text{to}-3.77\right)\times \text{Finger}–\text{ring test}\;\\ \;\left(1=“\text{Smaller}”\;\text{or}\;0=“\text{Just}\;\mathrm{fit}\;\text{or}\;\text{Larger}”\right) \end{array}$$

The estimation models for CCr_24_ are presented in Table S1.

### 3.4 Estimation Performance for Continuous Outcomes

**FIGURE 1:**
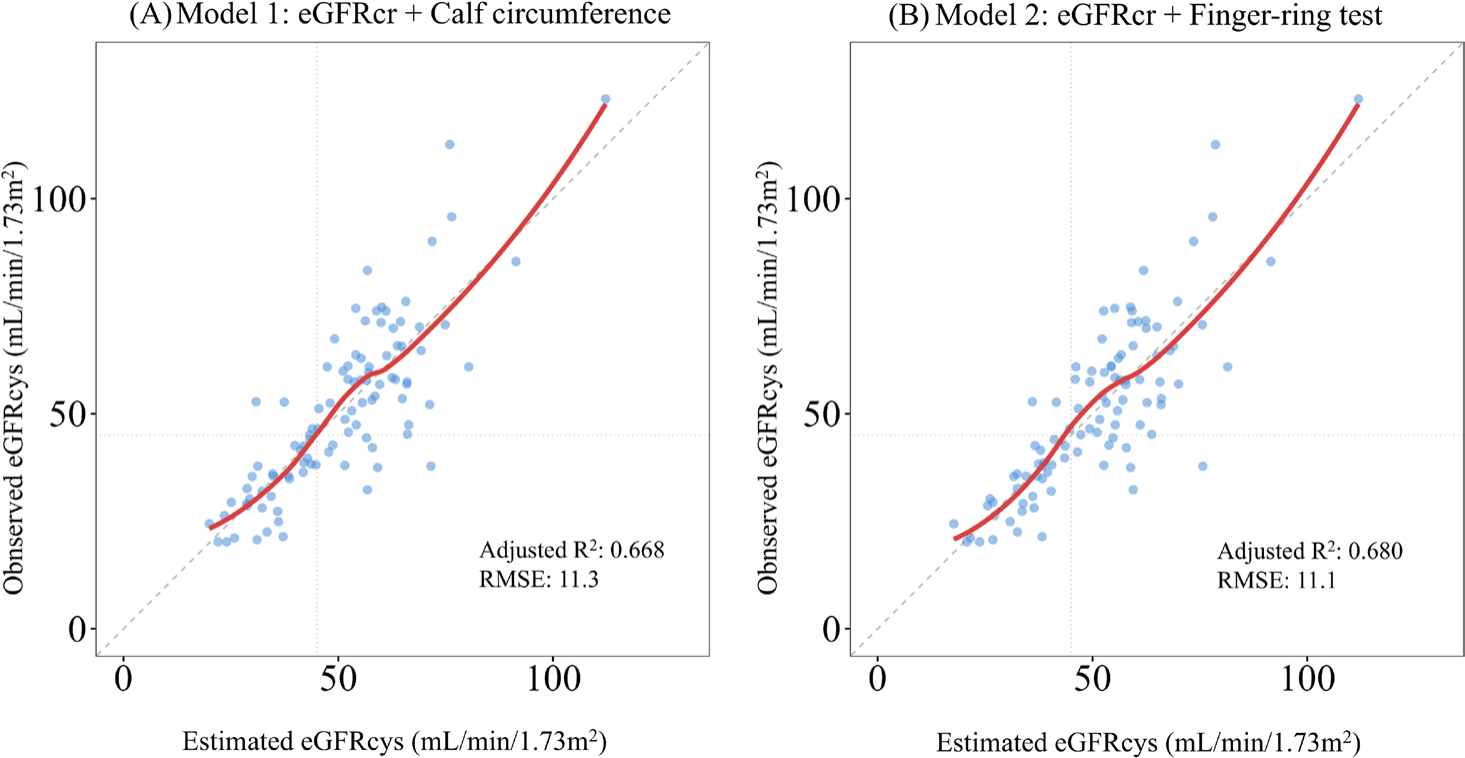
Calibration plots of estimated versus observed eGFRcys. Observed versus estimated eGFRcys values for the two extended estimation models are presented. Panel A shows Model 1 (eGFRcr + Calf circumference), and Panel B shows Model 2 (eGFRcr + Finger‐ring test). Each point represents an individual participant. The diagonal dashed line indicates perfect agreement between the estimated and observed values. The red line shows the locally weighted smoothing (LOESS) calibration curve. The model performance is summarized by the adjusted *R* ^2^ and root mean square error (RMSE) shown within each panel. eGFRcr, creatinine‐based estimated glomerular filtration rate; eGFRcys, cystatin C–based estimated glomerular filtration rate; RMSE, root mean square error.

For the continuous estimation of eGFRcys, the Basic model showed an adjusted *R* ^2^ of 0.641 and RMSE of 11.8. Model fit improved after adding muscle‐related indicators, with adjusted *R* ^2^ increasing to 0.668 (RMSE 11.3) in Model 1 and 0.680 (RMSE 11.1) in Model 2. Calibration plots demonstrated good agreement between estimated and observed values (Figure 1). Bootstrap‐corrected calibration slopes were 1.00, 1.01, and 1.01, respectively, with CITL values close to zero. Similar findings were observed for CCr_24_ (Figure S1).

### 3.5 Association With Reduced Kidney Function

For eGFRcys < 45 mL/min/1.73 m^2^, higher eGFRcr was associated with lower odds of reduced kidney function (OR 0.89; 95% CI 0.85 to 0.93; *p* < 0.001). Calf circumference was independently associated with lower odds (OR 0.69; 95% CI 0.54 to 0.85; *p* = 0.001), whereas a “Smaller” finger‐ring test result was associated with higher odds (OR 9.82; 95% CI 2.75 to 44.23; *p* = 0.001). Similar associations were observed for CCr_24_ < 45 mL/min/1.73 m^2^.

**FIGURE 2:**
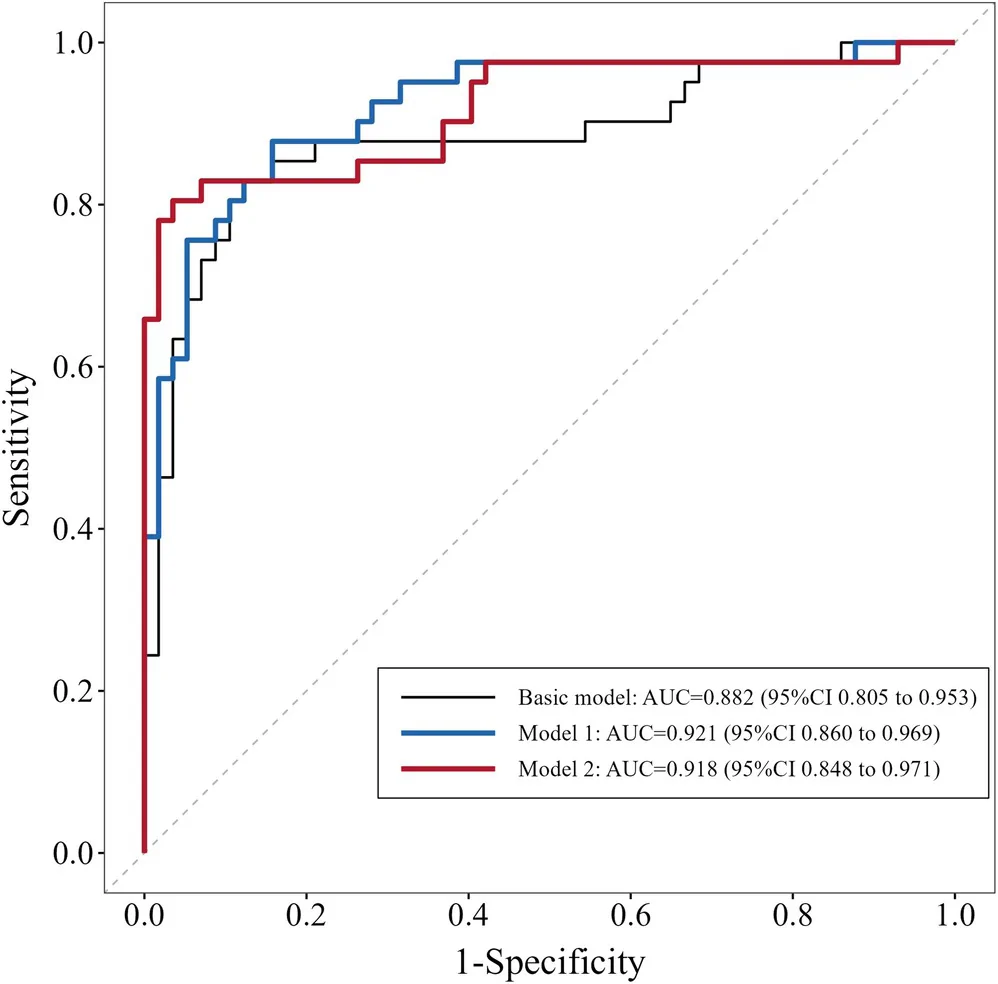
Receiver operating characteristic (ROC) curves comparing discrimination of the Basic model, Model 1, and Model 2 for identifying reduced kidney function defined as eGFRcys < 45 mL/min/1.73 m^2^. ROC curves were used to compare the discrimination of the Basic model (eGFRcr only), Model 1 (eGFRcr + Calf circumference), and Model 2 (eGFRcr + Finger‐ring test). The area under the curve (AUC) with a 95% confidence interval is shown. Differences in AUC were assessed using the DeLong test. AUC, area under the curve; CI, confidence interval; eGFRcys, cystatin C–based estimated glomerular filtration rate.

ROC analysis showed higher discrimination after the addition of muscle‐related indicators. For eGFRcys < 45 mL/min/1.73 m^2^ (Figure 2), AUC increased from 0.882 (95% CI 0.807 to 0.957) in the Basic model to 0.921 (95% CI 0.864 to 0.978) in Model 1 and 0.918 (95% CI 0.857 to 0.980) in Model 2, although differences were not statistically significant (DeLong *p* = 0.090 and 0.076, respectively). Optimism‐corrected Brier scores improved from 0.134 to 0.115 and 0.112. Similar results were observed for CCr24 < 45 mL/min/1.73 m^2^, with AUCs of 0.857, 0.905, and 0.912, respectively. The improvement reached statistical significance only for Model 2 (DeLong *p* = 0.037). Corresponding Brier scores decreased from 0.157 to 0.134 and 0.129.

Cross‐classification analysis (Table 2) revealed that among participants with eGFRcys < 45 mL/min/1.73 m^2^ (*n* = 41), eGFRcr alone correctly classified 56.1% (23/41), whereas both extended models correctly classified 80.5% (33/41). Among participants with eGFRcys ≥ 45 mL/min/1.73 m^2^ (*n* = 57), correct classification remained high (96.5%, 93.0%, and 94.7%, respectively). Similar findings were observed for CCr_24_, with correct classification increasing for participants with true CCr_24_ < 45 mL/min/1.73 m^2^ (*n* = 46), increasing from 56.5% (26/46) in eGFRcr alone to 69.6% (32/46) in Model 1 and 73.9% (34/46) in Model 2.

**TABLE 2:** Cross‐classification of estimated and observed kidney function categories defined by eGFRcys (< 45 vs. ≥ 45 mL/min/1.73 m^2^).

	True eGFRcys < 45 (*n* = 41)	True eGFRcys ≥ 45 (*n* = 57)
eGFRcr alone		
Estimated < 45	**23 (56.1%)**	2 (3.5%)
Estimated ≥ 45	18 (43.9%)	**55 (96.5%)**
Model 1 (eGFRcr + calf circumference)		
Estimated < 45	**33 (80.5%)**	4 (7.0%)
Estimated ≥ 45	8 (19.5%)	**53 (93.0%)**
Model 2 (eGFRcr + Finger‐ring test)		
Estimated < 45	**33 (80.5%)**	3 (5.3%)
Estimated ≥ 45	8 (19.5%)	**54 (94.7%)**

*Note:* Data are presented as *n* (column %). Estimated categories were defined using the fixed clinical threshold of 45 mL/min/1.73 m^2^, corresponding to CKD stage G3b. Bold values indicate correct classifications of participants within each observed kidney function category.

Abbreviations: CKD, chronic kidney disease; eGFRcr, creatinine‐based estimated glomerular filtration rate; eGFRcys, cystatin C‐based estimated glomerular filtration rate.

## 4 Discussion

This multicenter cross‐sectional study showed that incorporating simple bedside indicators associated with muscle mass, namely calf circumference and the finger‐ring test, into creatinine‐based assessment improved the identification of reduced kidney function in hospitalized older adults. Although improvements in overall model performance were modest, the proportion of patients with eGFRcys < 45 mL/min/1.73 m^2^ who were correctly identified increased from 56.1% with eGFRcr alone to 80.5% with each extended model. These findings suggest that bedside physical assessment may help identify patients in whom creatinine‐based estimates overestimate kidney function because of reduced muscle mass.

Unlike previous studies that primarily aimed to improve the numerical accuracy of GFR estimation, our study focused on identifying older adults with concealed renal impairment. This distinction is clinically relevant because reduced muscle mass may cause eGFRcr to overestimate kidney function, whereas practical bedside approaches to recognize such patients remain limited. Our findings suggest that calf circumference and the finger‐ring test may provide complementary information to eGFRcr, particularly when cystatin C measurement is unavailable or delayed. Although the improvements in AUC were not statistically significant, the higher classification rates observed with both extended models suggest that these bedside assessments provide additional information beyond GFRcr alone.

A major strength of this study is the use of simple bedside measures that require no specialist equipment, imaging, or additional laboratory testing. Their simplicity supports implementation in routine geriatric practice, particularly where cystatin C measurement is not routinely available [9, 27]. The multicenter design and bootstrap internal validation further strengthen the robustness of our findings. These measures may therefore provide a practical adjunct to eGFRcr when additional assessment of kidney function is warranted. This may be particularly relevant when prescribing renally cleared medications because overestimation of kidney function has been associated with adverse drug reactions in hospitalized older adults [28], and inappropriate prescribing remains common in patients with renal impairment [29]. Current guidelines recommend careful assessment of kidney function before prescribing renally cleared drugs [7, 21], yet reliance on eGFRcr alone may provide falsely reassuring estimates. Another strength is the use of eGFRcys as the reference outcome. The use of eGFRcys as the primary outcome is supported by current clinical recommendations and previous evidence in older adults. Although measured GFR using exogenous filtration markers remains the reference standard, it is rarely feasible in routine practice. Current KDIGO guidelines recommend cystatin C when creatinine‐based estimation is likely to be unreliable because of reduced muscle mass [7]. Furthermore, evidence from the Berlin Initiative Study and related investigations demonstrated that, among older adults with lower eGFRcys than eGFRcr, cystatin C‐based estimation showed closer agreement with measured GFR than creatinine‐based estimation [8, 12]. Because all participants in our cohort showed lower eGFRcys than eGFRcr, these findings further supported the use of eGFRcys as a clinically relevant comparator for evaluating concealed renal impairment in this study.

Several limitations should also be acknowledged. First, this study included only 98 hospitalized older adults from four community hospitals, limiting statistical power and generalizability. Second, the cross‐sectional design evaluated concurrent identification of reduced kidney function rather than prediction of future renal outcomes. Third, although eGFRcys is less influenced by muscle mass than creatinine, it remains an estimated rather than directly measured GFR and may be affected by non‐GFR determinants such as inflammation or thyroid dysfunction. Fourth, calf circumference and the finger‐ring test are surrogate indicators rather than direct measures of muscle mass and may also be influenced by body composition or residual fluid status despite excluding patients with severe edema. Finally, external validation is required before these models can be recommended for routine clinical application.

To facilitate clinical use, a simplified quick‐reference chart derived from the estimation equation may help clinicians approximate eGFRcys using routinely available eGFRcr values and bedside muscle‐related indicators (Table S2). However, this tool should be regarded as hypothesis‐generating until externally validated. Rather than replacing current kidney function assessment, bedside muscle‐related indicators may complement eGFRcr interpretation by helping identify patients who could benefit from confirmatory cystatin C measurement or more cautious prescribing of renally cleared medications.

## 5 Conclusion

In this multicenter cross‐sectional study, incorporating simple bedside muscle‐related indicators into creatinine‐based assessment improved the identification of reduced kidney function, defined by eGFRcys, in hospitalized older adults. These findings support the potential role of pragmatic bedside assessments as complementary information for interpreting creatinine‐based kidney function estimates in older adults at risk of reduced muscle mass. External validation and prospective studies are needed to determine whether this approach improves clinical decision‐making and patient outcomes.

## Author Contributions

Conception and design: S.M., K.‐e.S., M.K., N.D., and S.S. Analysis and interpretation of data: S.M. and K.‐e.S. Manuscript writing: S.M. and K.‐e.S. Approval of the final manuscript: S.M., K.‐e.S., M.K., N.D., K.F., and S.S.

## Funding

This study was supported by the Kochi Prefecture Clinical Research Fellowship, funded by donations from the Kochi Prefecture. Additionally, this research was supported by the charitable trust “Fund of Life (Inochi)” by the Kochi Shimbun and Kochi Broadcasting. The funders had no role in the study design, analyses, interpretation of the data, writing of the manuscript, or decision to submit the manuscript for publication.

## Ethics Statement

The study protocol was approved by the Institutional Ethics Review Board of Kochi University (Approval No: 2023‐95), and written informed consent was obtained from all participants.

## Conflicts of Interest

The authors declare no conflicts of interest.

## Supporting information


**Table S1:** Estimation models for the secondary outcome (CCr_24_).
**Table S2:** Quick reference chart for estimated eGFRcys (Model 2).
**Figure S1:** Calibration plots of estimated versus observed 24‐h creatinine clearance (CCr_24_).
**Figure S2:** Receiver operating characteristic (ROC) curves comparing discrimination of the Basic model, Model 1, and Model 2 for identifying reduced kidney function defined as CCr_24_ < 45 mL/min/1.73 m^2^.

## Data Availability

The data that support the findings of this study are available on request from the corresponding author. The data are not publicly available due to privacy or ethical restrictions.
