# Supplementary material for: Simple Bedside Muscle‐Related Indicators Improve the Identification of Reduced Kidney Function in Hospitalized Older Adults: A Multicenter Cross‐Sectional Study
Source: Geriatr Gerontol Int. 2026 Sep 24;26(10):e70851. doi: 10.1111/ggi.70851 (PMC13601913; doi:10.1111/ggi.70851)
Supplement: Supplementary file 1 [file GGI-26-0-s001.docx]

**Supplementary materials**

**Table S1. Estimation models for the secondary outcome (CCr_24_)**

| **Model** | **Estimation equation** |
| --- | --- |
| **Basic model** (eGFRcr only) | Estimated CCr_24_ = 9.73 (95% CI −0.24 to 19.71) +  0.68 (0.52 to 0.84) × eGFR_cr_ |
| **Model 1** (eGFRcr +Calf circumference) | Estimated CCr_24_ = −49.93 (95% CI −78.46 to −21.38) +  0.69 (0.54 to 0.84) × eGFR_cr_ + 1.92 (1.05 to 2.79) ×  calf circumference (per 1 cm) |
| **Model 2** (eGFRcr + Finger-ring test) | Estimated CCr_24_ = 17.88 (95% CI 7.96 to 27.80) +  0.69 (0.54 to 0.83) × eGFR_cr_ − 13.52 (−19.78 to −7.72) × finger-ring test (1 = “Smaller”; 0 = “just fit or Larger) |

Note: Values in parentheses are 95% confidence intervals. GFR values are expressed in mL/min/1.73 m^2^, and calf circumference in cm. The finger-ring test is coded as 1 (“Smaller”) or 0 (“Just fit” or “Larger”).

Abbreviations: CCr_24_, 24-hour urinary creatinine clearance; eGFR_cr_, creatinine-based estimated glomerular filtration rate; CI, confidence interval.

**Table S2. Quick reference chart for estimated eGFRcys (Model 2)**

Estimated eGFRcys (Smaller) = 10.74 + 0.77 × eGFRcr − 8.40,

Estimated eGFRcys (Just fit or Larger) = 10.74 + 0.77 × eGFRcr

|  | Estimated eGFRcys (mL/min/1.73m^2^) | |
| --- | --- | --- |
|  | **Finger-ring test** | |
| eGFRcr (mL/min/1.73m^2^) | **Smaller** | **Just fit or Larger** |
| 30 | 25 | 33 |
| 40 | 33 | 41 |
| 50 | 41 | 50 |
| 60 | 49 | 57 |
| 70 | 56 | 65 |
| 80 | 64 | 72 |
| 90 | 72 | 80 |

Abbreviations: eGFRcr, creatinine-based estimated glomerular filtration rate; eGFRcys, cystatin C-based estimated glomerular filtration rate

**Figure S1.**

**Figure S2.**

**Figure legends**

**Supplementary Figure S1. Calibration plots of estimated versus observed 24-hour creatinine clearance (CCr_24_).**

The observed and estimated CCr_24_ values are presented for the two extended estimation models. Panel A shows Model 1 (eGFRcr + Calf circumference), and Panel B shows Model 2 (eGFRcr + Finger-ring test). Each point represents an individual participant. The diagonal dashed line indicates perfect agreement between the estimated and observed values. The red line shows the locally weighted smoothing (LOESS) calibration curve. The model performance is summarized by the adjusted R² and root mean square error (RMSE) shown within each panel.

Abbreviations: CCr_24_, 24-hour creatinine clearance; eGFRcr, creatinine-based estimated glomerular filtration rate; RMSE, root mean square error.

**Supplementary Figure S2. Receiver operating characteristic (ROC) curves comparing discrimination of the Basic model, Model 1, and Model 2 for identifying reduced kidney function defined as CCr_24_ < 45 mL/min/1.73 m².**

ROC curves were used to compare the discrimination of the Basic model (eGFRcr only), Model 1 (eGFRcr + calf circumference), and Model 2 (eGFRcr + finger-ring test). The area under the curve (AUC) with 95% confidence interval is shown. Differences between the models were assessed using the DeLong test.

Abbreviations: AUC, area under the curve; CI, confidence interval; CCr_24_, 24-hour creatinine clearance.
